# c-Met-integrin cooperation: Mechanisms, tumorigenic effects, and therapeutic relevance

**DOI:** 10.3389/fcell.2022.994528

**Published:** 2022-10-14

**Authors:** Justas Stanislovas, Stéphanie Kermorgant

**Affiliations:** Spatial Signalling Group, John Vane Science Centre, Barts Cancer Institute, Queen Mary University of London, London, United Kingdom

**Keywords:** c-Met, integrins, signalling, trafficking, endocytosis, cancer, metastasis

## Abstract

c-Met is a receptor tyrosine kinase which upon activation by its ligand, the hepatocyte growth factor, mediates many important signalling pathways that regulate cellular functions such as survival, proliferation, and migration. Its oncogenic and tumorigenic signalling mechanisms, greatly contributing to cancer development and progression, are well documented. Integrins, heterogeneous adhesion receptors which facilitate cell-extracellular matrix interactions, are important in biomechanically sensitive cell adhesion and motility but also modulate diverse cell behaviour. Here we review the studies which reported cooperation between c-Met and several integrins, particularly β1 and β4, in various cell models including many tumour cell types. From the various experimental models and results analysed, we propose that c-Met-integrin cooperation occurs *via* inside-out or outside-in signalling. Thus, either c-Met activation triggers integrin activation and cell adhesion or integrin adhesion to its extracellular ligand triggers c-Met activation. These two modes of cooperation require the adhesive function of integrins and mostly lead to cell migration and invasion. In a third, less conventional, mode of cooperation, the integrin plays the role of a signalling adaptor for c-Met, independently from its adhesive property, leading to anchorage independent survival. Recent studies have revealed the influence of endocytic trafficking in c-Met-integrin cooperation including the adaptor function of integrin occurring on endomembranes, triggering an inside-in signalling, believed to promote survival of metastatic cells. We present the evidence of the cooperation *in vivo* and in human tissues and highlight its therapeutic relevance. A better understanding of the mechanisms regulating c-Met-integrin cooperation in cancer progression could lead to the design of new therapies targeting this cooperation, providing more effective therapeutic approaches than c-Met or integrin inhibitors as monotherapies used in the clinic.

## 1 Introduction

### 1.1 The tyrosine kinase receptor c-Met

**FIGURE 1:**
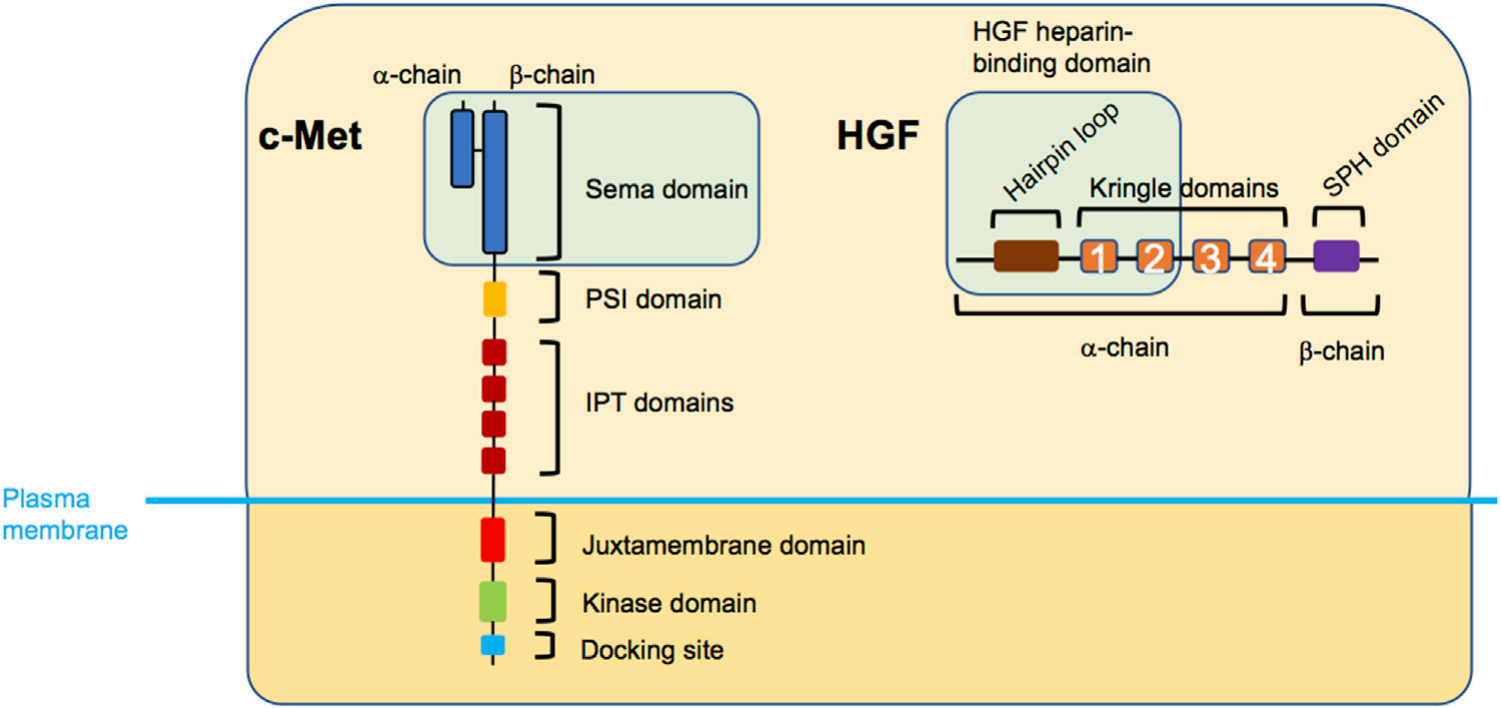
Structure of c-Met and HGF. The extracellular region of c-Met entails the Sema domain, plexin-semaphorin-integrin (PSI) domain, and four consecutively arranged immunoglobulin-plexin-transcription (IPT) factors domains, while the intracellular part is composed of a juxtamembrane domain, a kinase domain, and the docking site domain. HGF entails the hairpin loop, four consecutive kringle domains, and a serine protease homology (SPH) domain. The green boxes indicate the c-Met-HGF binding regions. Note, that the PSI and IPT domains are also involved in correct positioning of HGF.

**FIGURE 2:**
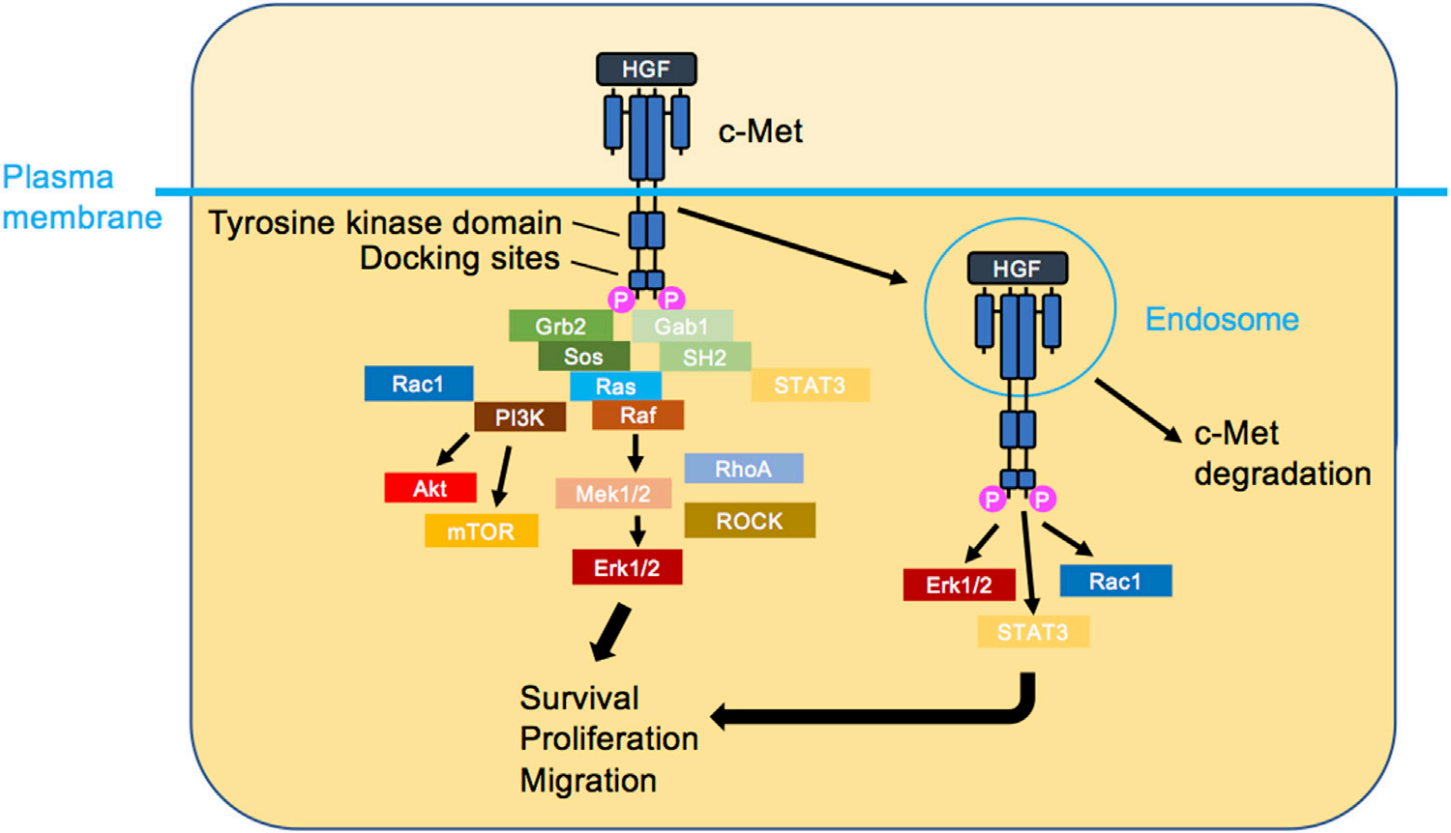
c-Met signalling. HGF triggers c-Met dimerisation, activation and the initiation of several signalling cascades. The classical view is that c-Met signalling occurs at the plasma membrane (left). Recently, it has been shown that HGF-bound c-Met internalises rapidly and transmits signalling from endosomes (right), leading to the activation of signal transducer and activator of transcription 3 (STAT3), ERK1/2 and PI3K/AKT. Ultimately, cellular responses such as survival, proliferation and migration are induced.

c-Met protooncogene-encoded c-Met protein (also referred to as Met/MET/c-MET) is a receptor tyrosine kinase mostly expressed by epithelial cells in a range of tissues (Gherardi et al., 2012). Structurally, mature c-Met is an αβ heterodimer composed of extracellular N-terminal tail, Sema domain which facilitates ligand binding, plexin-semaphorin-integrin domain, and four consecutively arranged immunoglobulin-plexin-transcription factors domains (Figure 1). The intracellular region following the transmembrane part entails a regulatory juxtamembrane domain, catalytic kinase domain, and a multi-substrate docking site followed by the C-terminus (Park et al., 1987; Liu, 1998; Stamos et al., 2004; Gherardi et al., 2003; Kozlov et al., 2004; Longati et al., 1994; Ponzetto et al., 1994). c-Met, activated by its ligand hepatocyte growth factor (HGF) (Stoker et al., 1987; Nakamura et al., 1989; Weidner et al., 1991), plays a fundamental role in embryogenic, morphogenic and physiologic responses (Trusolino et al., 2010). HGF, also an αβ heterodimer in its mature form, is composed of the N-terminus, a hairpin loop, four consecutive kringle domains, all housed by the α-subunit, and a serine protease homology domain, housed by the β-subunit. Typically, HGF binding to c-Met’s Sema domain is enhanced *via* the HGF heparin binding domain (hairpin loop and the first and second kringle domains) (Gherardi et al., 2006; Naldini et al., 1992; Nakamura et al., 1989; Lokker et al., 1992; Mizuno et al., 1994; Kong-Beltran et al., 2004). For an in-depth investigation of HGF-c-Met binding and its 3-dimensional (3D) depiction, refer to a recent structural study (Uchikawa et al., 2021). HGF/c-Met axis activates signalling cascades regulating cellular functions such as survival, proliferation and motility. HGF-induced c-Met homodimerization and resultant kinase domain activation allows tyrosine (Y) phosphorylation of the docking sites Y1349 and Y1356, enabling formation of the signalling complexes (Ponzetto et al., 1994) (Figure 2). Activated c-Met induces intracellular downstream signalling such as the phosphoinositide 3-kinase (PI3K) and mitogen activated protein kinase (MAPK) pathways and can activate the transcription factor signal transducer and activator of transcription 3 (Trusolino et al., 2010). Another consequence of HGF binding to c-Met is a rapid endocytosis of HGF-bound and activated c-Met, often through the classical clathrin and dynamin-dependent pathway (Hammond et al., 2001; Kermorgant et al., 2003). Interestingly, c-Met remains bound to HGF and able to signal on endosomes (Kermorgant et al., 2004; Kermorgant and Parker, 2008) prior to its progressive degradation (Kermorgant et al., 2003). Depending on the situation, a proportion of c-Met can be recycled to the plasma membrane instead (Joffre et al., 2011; Parachoniak et al., 2011). Moreover, we have reported that c-Met “endosomal signalling” is required for HGF-dependent cell migration (Kermorgant et al., 2004; Kermorgant and Parker, 2008) and c-Met oncogenicity *in vitro* and *in vivo* (Joffre et al., 2011). Furthermore, c-Met activates distinct signalling cascades depending on which endosome it is localized, fine-tuning functional outcomes (Barrow-Mcgee and Kermorgant, 2014; Menard et al., 2014; Barrow-Mcgee et al., 2016; Hervieu et al., 2020). Given the role of c-Met signalling in governing cell behaviour, its deregulation is believed to be robustly exploited by cancerous cells. High level of HGF in the tumour microenvironment favouring paracrine tumour-stroma crosstalk, c-Met overexpression, ligand-independent activation, exon 14 deletion and less often kinase domain mutations are well documented mechanisms utilised by the tumour cells (Comoglio et al., 2018; Baldacci et al., 2018; Duplaquet et al., 2018). Resultant enhancement of their survival, proliferative and motile abilities, contributes to tumorigenesis and poor disease outcomes. Further, c-Met may be a strong mediator of resistance to targeted therapy as well as chemotherapy agents (Engelman et al., 2007; Corso and Giordano, 2013; Fernandes et al., 2021; Wood et al., 2021). C-Met is therefore a major target for cancer therapy with many inhibitors (mostly tyrosine kinase inhibitors or antibodies) developed or in development (Gherardi et al., 2012; Huang et al., 2020).

### 1.2 The integrin adhesion receptors

#### 1.2.1 Integrin structure

**FIGURE 3:**
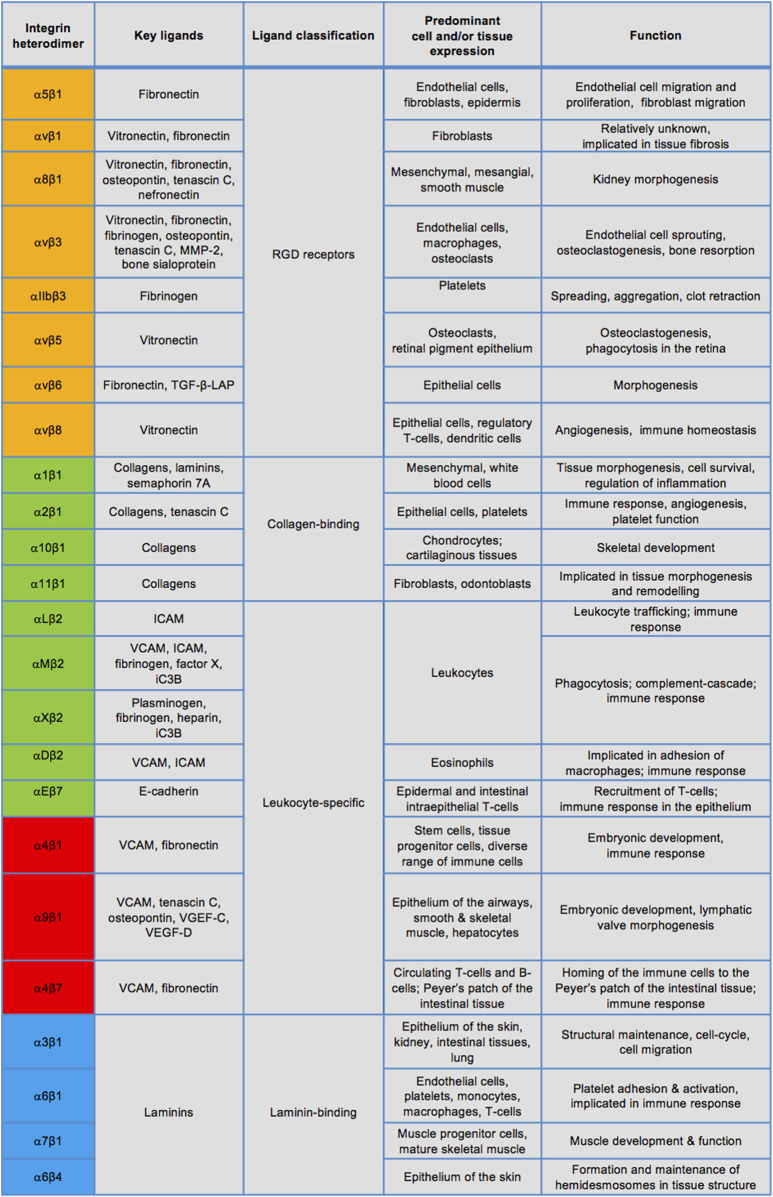
Known integrin heterodimers and their ligands, expression, and physiological roles. Integrins in the orange coloured rows have an α subunit evolutionarily related to PS2 proteins of *Drosophila*, those in the green coloured rows share an α subunit with an αI domain. The blue rows indicate integrins containing an α subunit related to PS1 *Drosophila* proteins and many of these integrins recognise the ECM ligands containing the RGD motif, whereas the red rows denote integrins separated by their α4/α9 domains. The list of ligands, cell/tissue expression and functions is not exclusive. MMP-2: matrix metalloprotease-2, TGF-β-LAP: transforming growth factor- β-latency-associated peptide, ICAM: intracellular adhesion molecule, VCAM: vascular cell adhesion molecule.

Integrins are a large family of cell-surface adhesion receptors, which are fundamental in facilitating interactions between cells and their extracellular matrix (ECM) (Hynes, 2002; Hynes, 2004). Thus, ECM proteins, such as collagen, laminin or fibronectin, act as integrin ligands, while the intracellular integrin region connects to the actin cytoskeleton. Integrins are a type I transmembrane proteins which exist in 24 known heterodimeric combinations of their α and β subunits, of which there are 18 and eight identified types, respectively (Figure 3). Integrins can be divided into four groups by their evolutionary α -subunit similarity and largely commonly shared ligands (laminin-binding, collagen-binding, arginine-glycine-aspartic acid (RGD) motif-binding, and leukocyte-specific) (Johnson et al., 2009). Specific ECM ligand residue sequences, such as the RGD motif, facilitating integrin-ECM ligand binding, may involve both α and β monomers. This was for example demonstrated for αvβ3 binding to fibronectin, fibrinogen and vitronectin (Takagi et al., 2002; Xiong et al., 2002). Some integrins, such as α2β1 which binds to the collagen proteins, exhibit ECM ligand binding domains solely on the α-subunit (Emsley et al., 2000). Importantly, while heterogeneity of the heterodimers allows binding specificity and therefore diverse functional effects, many integrins can redundantly bind the same ECM ligand (Johnson et al., 2009). The less ligand-specific integrins, however, may exhibit different functional output dependent on the biochemical and mechanical regulation, and cooperation with other cell-surface molecules (Kechagia et al., 2019).

#### 1.2.2 Integrin activation

**FIGURE 4:**
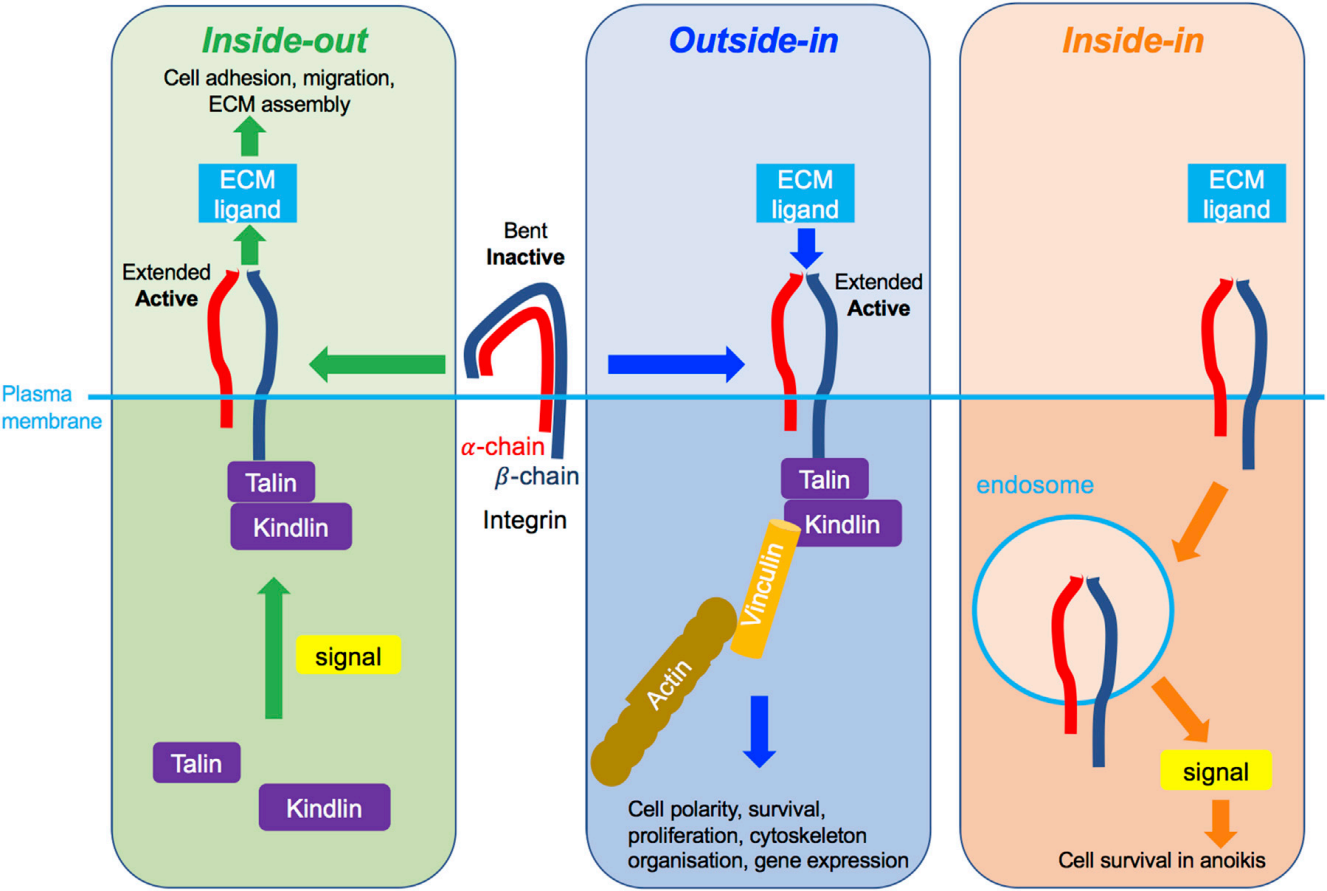
Different modes of integrin signalling. Left green box: Inside-out integrin signalling. An intracellular signal, such as promoted by an activated growth factor receptor, triggers the recruitment to the integrin β-chain of key molecules including talin and kindlin, leading to conformational change of integrins, which become extended and active and can then bind to its ligand in the ECM. As a consequence, cell adhesion, migration and/or ECM assembly are promoted. Middle blue box: Outside-in integrin signalling. The binding of the integrin to the ECM outside of the cell triggers its change of conformation to become extended and active, leading to the recruitment of several molecules to the cytoplasmic tail of the β-chain, such as talin, kindlin and vinculin, and the connection to the actin cytoskeleton. As a consequence, cell polarity, survival, cytoskeleton organisation or gene expression are promoted. Right organge box: Inside-in signalling, a third mode of integrin signalling, recently described for β1 integrin. The integrin transmits signalling from endosomes in cells in suspension alone. As a consequence, cell survival in anoikis is promoted.

Three modes of integrin activation have been described and may not be mutually exclusive. Integrin activation may occur *via inside-out* signalling (Figure 4, left panel), whereby an intracellular signal, for example transduced by a growth factor receptor (GFR), stimulates the recruitment of intracellular adaptor proteins, such as the necessary actin-binding proteins talin and kindlin (Theodosiou et al., 2016; Sun et al., 2019), and their consequent binding to the cytosolic integrin domain. This in turn facilitates conformational change of the extracellular integrin region from the functionally inactive “bent-closed” to the “extended-open” conformation with high ligand binding affinity (Shattil et al., 2010; Kim et al., 2011). This as a result enables integrin-ECM ligand adhesion. In the cytoplasmic tail of β integrins some key tyrosine residues get phosphorylated and their mutations interfere with talin binding, integrin activation, integrin-mediated adhesion and downstream signalling (Calderwood et al., 2002). In most integrins, these tyrosines are within the highly conserved NPXY/NXXY motifs and act as docking sites allowing the recruitment of signalling molecules such as focal adhesion kinase (FAK), integrin-linked kinase (ILK) and Src kinase (Calderwood et al., 2002; Anthis et al., 2009). Shc adaptor protein was found to bind these phosphorylated tyrosines and enable FAK phosphorylation (Tahiliani et al., 1997; Dans et al., 2001). This in turn induces phosphorylation of downstream signalling targets, such as the extracellular-related kinase 1/2 (ERK1/2), an effector of the MAPK signalling pathway.

The *outside-in* signalling (Figure 4, middle panel), on the other hand, is believed to be initiated by the ECM ligand binding to the extracellular region of its cognate integrin receptor, inducing a conformational change of the integrin from the “bent-closed” to the elongated, “extended open” conformation. Moreover, ECM-generated mechanical force may also promote active integrin conformation. This as a result ultimately enables adaptor protein association with the cytosolic integrin domain, assembly of the signalosome and initiation of the downstream signalling. *Outside-in* integrin signalling is also described by the recruitment of FAK/Src kinases and activation of downstream signalling cascades, notably PI3K and Ras small guanosine triphosphatase (GTPase)-MAPK pathways (Ivaska and Heino, 2011).

Further, more recently, a third mode of integrin signalling has been described, named *inside-in* signalling (Barrow-Mcgee et al., 2016) (Figure 4, right panel), whereby internalised integrins promote signalling from the endomembranes (Alanko et al., 2015; Barrow-Mcgee et al., 2016). *Inside-in* signalling has been described for the β1 integrin using several different cell lines, notably telomerase-immortalised foreskin fibroblasts, mouse-embryonic fibroblasts, breast cancer MDA-MB-231, MDA-MB-468, and lung cancer NCI-H460 and A549 cells (Alanko et al., 2015; Barrow-Mcgee et al., 2016). There is limited data on the signals associated with the inside-in signalling. Thus far phosphorylated FAK (Alanko et al., 2015) and ERK1/2 (Barrow-Mcgee et al., 2016) have been detected on active β1-containing early endosomes (Alanko et al., 2015) and autophagy related endomembranes (ARE) (Barrow-Mcgee et al., 2016) respectively. Moreover, inhibition of integrin endocytosis using dynamin inhibition and/or Rab21 siRNA reduces FAK or ERK1/2 phosphorylation, suggesting integrin can signal on the endomembranes (Alanko et al., 2015; Barrow-Mcgee et al., 2016). Functionally, this integrin inside-in signalling was shown to protect detached cells against anoikis, allowing them to metastasise more efficiently (Mai et al., 2014; Alanko et al., 2015; Barrow-Mcgee et al., 2016). Inside-in signalling is further discussed in Section 4.2 in relation to c-Met.

#### 1.2.3 Integrin trafficking

Integrin signalling is spatiotemporally regulated through dynamic endocytic internalisation, recycling and degradation. Integrins are endocytosed predominantly through clathrin-dependent and clathrin-independent, such as caveolin-dependent, mechanisms. Rab and ARF subfamilies of Ras GTPase protein superfamily are particularly important in integrin trafficking (Bridgewater et al., 2012; De Franceschi et al., 2015). Rab5-positive early endosomes and Rab4-positive recycling endosomes are the key compartments in recycling of the internalised integrins back to the plasma membrane (Moreno-Layseca et al., 2019). This constantly cyclic state of integrins at the plasma membrane is important in the turnover of focal adhesion complexes and thus in part regulates cellular response.

#### 1.2.4 Integrin function

Indeed the conventional function of integrins is to facilitate ECM ligand-generated signal transduction, allowing the cell to sense the biochemical and biomechanical dynamics of the extracellular environment and exhibit appropriate cellular response (Geiger and Yamada, 2011). Integrin-facilitated cell-ECM adhesomes are plastic and highly diverse molecular structures of varying mechanical strength, such as nascent adhesion complexes, most studied focal adhesions, and fibrillar adhesions. Although integrin-mediated signalling may regulate cell survival, differentiation and proliferation, early characterisation has established the particular importance of integrins in cell adhesion and motility (Huttenlocher and Horwitz, 2011). Particularly, focal adhesions are large complexes which incorporate clusters of integrins, structural adaptor molecules such as talin, paxillin and vinculin which link the integrins to the actin filaments, as well as downstream signalling molecules. Assembly and turnover of focal adhesions are key in mesenchymal cell migration, with the role of FAK and Src kinases in phosphorylating scaffold proteins such as paxillin. This in turn leads to small GTPase Rac1 activation *via* guanine exchange factor recruitment, enabling actin-related protein 2/3 complex activation and its-mediated actin polymerisation. The latter is responsible for driving cell protrusions. For example, α5β1 heterodimer, particularly important in the normal function of endothelium and *epidermis*, is a strongly specific receptor of fibronectin capable of activating FAK and Src kinases (Mitra and Schlaepfer, 2006; Caswell et al., 2007). α5β1 knockout (KO) is embryonically lethal due to the lack of and/or abnormal angiogenic development (Yang et al., 1993; Francis et al., 2002).

α6β4 integrin, on the other hand, through binding to the intracellular cytokeratin filaments and extracellular laminins of the basement membrane establishes the hemidesmosome complexes. Hemidesmosomes are important in tissue embryogenesis, cell polarity and wound healing through anchoring the epithelium to the basement membrane (Litjens et al., 2006; Walko et al., 2015; Stewart and O’connor, 2015). Both α5β1 and α6β4 are strongly implicated in pro-migratory, pro-invasive and metastatic tumour cell behaviour (Hou et al., 2020; Stewart and O’connor, 2015).

### 1.3 Integrin-growth factor receptor signal integration

Several studies have reported crosstalk between integrins and GFRs, particularly receptor tyrosine kinases. Early studies indicate αvβ3 association with vascular endothelial growth factor (VEGF) receptor and platelet derived growth factor receptor (Schneller et al., 1997; Soldi et al., 1999; Borges et al., 2000), whereas α6β4 shows integration with epidermal growth factor receptor and the ErbB2 receptor (Falcioni et al., 1997; Mariotti et al., 2001). Epidermal growth factor receptor also shows association with α5β1 (Miyamoto et al., 1995; Sieg et al., 2000), exhibiting epidermal growth factor receptor phosphorylation in an epidermal growth factor ligand-independent manner through αvβ3 and β1 clustering and transactivation (Moro et al., 1998). Collectively, these early studies implicate integrin-GFR association and cooperative signalling to promote cell survival, proliferation, migration, invasion, tumorigenic angiogenesis, and metastasis (Ivaska and Heino, 2011; Hamidi and Ivaska, 2018).

Here we review the studies which have reported c-Met and integrin cooperation and their role in cell migration, invasion, anchorage-independent growth and tumorigenesis. Through carefully analysing the experimental conditions and results of each publication, we propose that c-Met-integrin cooperation mainly occurs through the conventional inside-out or outside-in signalling mechanisms. Thus, either c-Met activation triggers integrin activation and cell adhesion or integrin adhesion to its extracellular ligand triggers c-Met activation. Furthermore, we also describe a less conventional mechanism in which an integrin acts as a c-Met signalling adaptor, which appears to be independent from its adhesive property. Recent studies have revealed the influence of endocytic trafficking in c-Met-integrin cooperation including the adaptor function of integrin occurring on an endosome following the co-trafficking of the two molecules, triggering an inside-in signalling. Although here we focus on the evidence pertaining to each mode of signalling cooperation, it is likely and indeed in some studies apparent that these types of cooperation are not mutually exclusive. We also present the evidence of the cooperation *in vivo* and in human tissues and highlight its therapeutic relevance.

## 2 c-Met-integrin cooperation *via* inside-out signalling

One form of cooperation appears to occur through the inside-out signalling, whereby c-Met, dependently or independently of HGF, acts upstream of the integrin receptor, inducing its activation and triggering downstream signalling and cell function.

### 2.1 Hepatocyte growth factor-dependent c-Met signalling upstream of integrins

Early studies reported that HGF stimulation of normal and cancer cells increased their adhesion to integrin ligands such as collagen, laminin-1, vitronectin, fibronectin or vascular cell adhesion molecule-1. Moreover, the adhesion was inhibited by relevant blocking antibodies of integrins, including αvβ3, and β1 and its partners α2, α3, α4, and α5 (Trusolino et al., 1998; Beviglia and Kramer, 1999; Weimar et al., 1997; Van Der Voort et al., 1999; Tjin et al., 2006). More recently, fibroblast-secreted HGF was shown to promote human lung adenocarcinoma cell adhesion on laminin-1 and their polarization in acini in 3D culture. These were blocked by an anti-β1 integrin antibody (Datta et al., 2017). Thus, although integrin specificity differs, similar adhesion-dependent HGF/c-Met axis-stimulated cell behaviour can be seen across different cell types.

Further studies reported the role of integrins in HGF-dependent cell migration. HGF triggered cell migration on laminin, the β4 ligand (Ephstein et al., 2013). Moreover, the inhibition of α9, α2β1, and α3β1 integrins with relevant specific blocking antibodies significantly reduced HGF-induced migration of lymphocytic endothelial cells and breast carcinoma cells (Beviglia and Kramer, 1999; Kajiya et al., 2005).

How c-Met controls the integrin activity in these HGF-dependent inside-out c-Met-integrin cooperation processes is not fully elucidated. One mechanism appears to be an increase in the integrin expression levels, assessed at the cell surface or as total expression level depending on the study, although mechanisms involved are unknown. Thus HGF triggers an increase in the expression of α9 integrin mRNA in lymphocytic endothelial cells (Kajiya et al., 2005). HGF also promotes expression of β1 protein, c-Met and ILK, as determined by immunohistochemistry and/or western blot, during HGF-dependent wound healing *in vitro* and *in vivo* (Li et al., 2013). ILK small-interfering RNA (siRNA) impaired HGF-mediated increase in β1 expression and wound healing which suggests a reciprocal ILK role (Li et al., 2013).

Another potential mechanism is an increase in integrin phosphorylation as observed for β4 integrin upon HGF stimulation (Franco et al., 2010).

A third potential mechanism is HGF-controlled integrin localisation. Thus, HGF induces β1 localisation to the basolateral membrane of human lung adenocarcinoma in 3D acini as shown using fluorescence-activated cell sorting (Datta et al., 2017). Downstream of β1 integrin, Src-p190A Rho-GTPase-activating-protein induced the inhibition of RhoA-Rho-associated coiled-coil kinase one signalling. The latter was required for the induction by HGF stimulation of acinar polarity in lung adenocarcinoma cells (Datta et al., 2017). Moreover, recent studies reported that HGF can control integrin intracellular trafficking. Thus, we and colleagues have shown that HGF stimulates a rapid internalisation of β1 integrin followed by recycling back at a later time. The loss of expression of the clathrin adapter huntingtin-interacting protein one by siRNA prevented HGF-dependent β1 integrin internalisation as well as mesenchymal and collective cell invasion from spheroids grown in 3D Matrigel (Mai et al., 2014). This suggests that c-Met promotes collective cell invasion *via* the stimulation of huntingtin-interacting protein 1-dependent β1 integrin trafficking, underscoring the role of endocytic processes in c-Met-integrin cooperation and its tumorigenic effect.

The KO of the Rho GTPase ARF6 in mice endothelial cells was shown to impair the HGF-dependent migration of these cells as well as their spread on and adhesion to the β1 integrin ligands collagen I and IV, and fibronectin. In parallel, ARF6 KO or short-hairpin RNA knockdown (KD) of its activator, the guanine exchange factor general receptor for 3-phosphoinositides 1, impaired HGF-stimulated β1 integrin recycling as shown using biotin-labelling and immunostaining, with no change in total β1 expression (Hongu et al., 2015). HGF-dependent *in vitro* angiogenesis was significantly reduced upon ARF6 siRNA-mediated KD or pharmacological inhibition of general receptor for 3-phosphoinositides 1. As tumour growth and associated angiogenesis were significantly reduced in conditional mice with ARF6 gene deletion in endothelial cells or pharmacological inhibition of general receptor for 3-phosphoinositides 1, this study therefore suggested that the effect of ARF6 on angiogenesis and tumour progression occurs *via* HGF-dependent β1 integrin recycling. Although KD of general receptor for 3-phosphoinositides one and other guanine exchange factors inhibited HGF-mediated β1 recycling (Hongu et al., 2015), the signalling cascade governing β1 trafficking downstream of HGF stimulation is unknown. Given the current literature, it is further unclear whether integrin expression and recycling are coupled.

### 2.2 Hepatocyte growth factor-independent c-Met signalling upstream of integrins

Fewer studies suggest that c-Met may also initiate integrin signalling without HGF stimulation. Thus, loss of c-Met expression *via* siRNA transfection in ovarian cancer cells, which overexpressed c-Met, triggered a reduction in β1 and α5 expression and reduced cell adhesion on fibronectin and vitronectin, and to the peritoneum of mice (Sawada et al., 2007).

c-Met silencing also triggered a decreased expression of β1 and α3 subunits in gastric cancer cells *in vitro* and, in parallel, their strongly reduced proliferation, invasion, adhesion, as well as reduced peritoneal dissemination *in vivo* (Wang et al., 2012). c-Met, although independently of its kinase activity, was also shown to be required for α3β1 expression and subsequent prevention of anoikis and promotion of survival of laminin-adherent primary prostate epithelial cells (Tesfay et al., 2016).

## 3 Integrin-c-Met cooperation *via* outside-in signalling

Early reports implicated that cell adhesion triggered c-Met activation in the absence of exogenous HGF (Wang et al., 1996; Wang et al., 2001). The results from these and further studies suggest c-Met-integrin cooperation can occur through an outside-in signalling, whereby an integrin activation, triggered by binding to its extracellular ligand in the ECM, thus promoting cell-surface adhesion, is also able to promote c-Met phosphorylation. Thus, treating or plating ovarian, breast, lung or prostate cancer cells with or on integrin substrates, such as fibronectin, collagen or laminin, was shown to trigger c-Met phosphorylation in various cell models (Mitra et al., 2011; Hui et al., 2009; Jahangiri et al., 2017; Singh et al., 2019; Sridhar and Miranti, 2006). c-Met phosphorylation was strongly reduced when carcinoma cells were cultured in suspension. It was recovered upon re-plating on fibronectin (Hui et al., 2009) or collagen (Wang et al., 2001). Further, increasing c-Met phosphorylation was observed upon plating cells on increasing concentrations of fibronectin (Jahangiri et al., 2017).

Moreover, siRNA-mediated silencing of integrins α5, α5β1, β1 or β5, or blocking antibodies of α5β1 or α5, or fibronectin siRNA reduced or abolished c-Met phosphorylation *in vitro* or in mice xenografts (Mitra et al., 2011; Ding et al., 2015; Huang et al., 2019a; Huang et al., 2019b; Singh et al., 2019; Chang et al., 2019). Addition of HGF, which triggered c-Met phosphorylation, was able to overcome the inhibition of ovarian cancer cell invasion by α5β1 blocking antibody or siRNA, demonstrating that integrin acts upstream of c-Met in an HGF-independent manner (Mitra et al., 2011).

The exact mechanisms of c-Met phosphorylation downstream of an integrin, however, are not clear. The cytoplasmic domain of integrin may play a role in c-Met phosphorylation. This was demonstrated by the expression of a truncated form of β4 integrin, β4-1355T, lacking the major tyrosine phosphorylation sites in the cytoplasmic tail, which led to the reduction in c-Met phosphorylation (Yoshioka et al., 2013). Depletion by short-hairpin RNA or inhibition of ILK were shown to block HGF-independent c-Met phosphorylation of fibronectin-adherent glioblastoma cells (Jahangiri et al., 2017). Another study reported that fibronectin adhesion-dependent c-Met phosphorylation was mediated by a cascade comprising FAK phosphorylation upstream of activated Src, FAK and Src occuring in a complex (Hui et al., 2009). Overall, Src-FAK pathway has been identified as a dominant signalling pathway downstream of integrin-activated c-Met (Mitra et al., 2011; Sridhar and Miranti, 2006; Jung et al., 2011).

## 4 c-Met-integrin cooperation *via* a non-adhesive signalling function of integrins

### 4.1 The role of integrins as c-Met signalling adaptor

Interestingly, some studies have shown that integrins can act as signalling adapters downstream of c-Met, enabling a signalling platform. This function appears to be independent from the adhesive property of integrins and was shown to lead to invasion (Trusolino et al., 2001) and anchorage-independent growth (Trusolino et al., 2001; Bertotti et al., 2005; Bertotti et al., 2006; Franco et al., 2010; Barrow-Mcgee et al., 2016).

Thus an early study reported that HGF stimulated β4 tyrosine phosphorylation, leading to the recruitment of p52Shc and PI3K to β4 (Trusolino et al., 2001). The use of c-Met mutants demonstrated that c-Met kinase activity was required. In this c-Met-β4 cooperation, the integrin was shown to play the role of an adaptor to amplify and sustain AKT and ERK1/2 phosphorylation downstream of c-Met. The binding of p52Shc to two specific tyrosines in the integrin cytoplasmic tail was shown to be required to connect the integrin to the signaling pathways (Trusolino et al., 2001). Functionally, β4 was reported to be necessary for HGF-induced c-Met-mediated cell invasion although independently of its adhesive integrin property. Indeed neither anti-β4 antibodies nor the expression of β4 lacking the extracellular domain blocked the invasion (Trusolino et al., 2001; Bertotti et al., 2005). Other molecules may also facilitate integrin-mediated signalling platform, as cluster of differentiation 151 (CD151) depletion led to the loss of β4 phosphorylation by c-Met, reduction in HGF-induced growth factor receptor bound protein 2-associated binding protein 1(Gab1) and growth factor receptor bound protein 2 (GRB2) association and ERK1/2 phosphorylation. CD151 depletion also impaired cell growth on soft agar and protection from anoikis (Franco et al., 2010).

We later reported that, in various human and mouse cells, including lung and breast cancer cells, β1 siRNA KD, KO or the expression of a β1 form mutated in its two NXXY domains, prevented the sustained phosphorylation of ERK1/2 stimulated by c-Met, when activated by HGF or constitutively by an oncogenic alteration. A similar pattern was observed for p52Shc phosphorylation, whereas c-Met phosphorylation was unaltered. Moreover, c-Met, β1 and p52Shc co-immunoprecipitation (co-IP) was increased upon c-Met activation. Furthermore, p52Shc siRNA KD impaired c-Met-β1 co-IP. These results suggested that β1, through its NXXY domain in the cytoplasmic tail, also functioned as an adapter linking c-Met to p52Shc, subsequently allowing sustained activation of ERK1/2. Importantly, this signalling occurred in adherent and detached cells, and β1, as well as its NXXY domain, were found to be required for c-Met-dependent anchorage growth in soft agar, protection against anoikis and *in vivo* tumorigenesis and invasion. Interestingly, while the adhesive property of integrin was shown to not be required for this c-Met-β1 cooperation, its active conformation appeared to promote the cooperation (Barrow-Mcgee et al., 2016).

### 4.2 Endosomal c-Met-integrin cooperation: Inside-in signalling

Furthermore, our study revealed that this c-Met-β1 signaling cooperation occurred on an endomembrane following co-internalisation of the two molecules (Barrow-Mcgee et al., 2016). Thus c-Met activity induced by HGF stimulation or oncogenic alterations triggered c-Met-β1 integrin co-internalisation which is dependent on clathrin expression and dynamin activity. This is followed by co-trafficking through the endomembranes which contain the autophagosome marker microtubule associated light-chain 3B. As our results suggested this endomembrane is distinct from the classical autophagosome and appeared to belong to a novel non-canonical autophagy pathway similar to the recently described LC3 Associated Phagocytosis (Florey, 2018), it was termed autophagy related endomembrane (ARE). Results obtained suggested that the adaptor function of β1, leading to sustained ERK1/2 signalling and increased anchorage-independent survival, occurs on ARE. Vesicular triple colocalisation of c-Met, β1, and microtubule associated light-chain 3B was observed upon c-Met activation. To reduce the lipidation of light-chain 3B, and thus the formation of ARE, we employed siRNA of autophagy-related gene five which led to the impairment of c-Met-dependent, sustained phosphorylation of ERK1/2 and p52Shc, anoikis protection and invasion *in vivo*. Similar phenotypes were seen with the loss of β1 (Barrow-Mcgee et al., 2016).

It was also shown that β1 integrin siRNA or NXXY domain mutant led to an impaired c-Met endocytosis (Barrow-Mcgee et al., 2016), indicating that the two molecules promote the trafficking of each other. Interestingly, c-Met and β1 integrin internalisation was reduced in cells expressing β1 integrin NXXY due to a decreased expression of Rab21. The effect of β1 integrin NXXY domain on c-Met endocytosis and on c-Met signalling on endosomes was decoupled through the overexpression of Rab21 in cells expressing β1 integrin NXXY domain. Although c-Met endocytosis was rescued, ERK1/2 sustained signalling was not, further suggesting a role for β1 integrin NXXY domain in sustaining c-Met signalling in addition to co-internalisation.

Cancer cells which metastasise need to survive mostly unanchored during their transit in the blood or lymphatic circulation. Thus, this inside-in c-Met-β1 integrin cooperation, which occurs in non-adherent cells and is independent of β1 adhesive property, could be used by the metastatic cancer cells during their transit in the blood or lymphatic circulation. There, integrin adhesion requirement is reduced while there is an enhanced need for sustained signalling to protect cells against anoikis.

Another recent study reported that two splice variants of the co-receptor neuropilin-1 increase c-Met and β1 integrin interaction and their co-internalisation and co-accumulation on endosomes. This provides persistent signals to activate the FAK/p130Cas pathway, thereby promoting colorectal cancer cell migration, invasion and metastasis (Huang et al., 2019b).

Hepatocellular carcinoma (HCC)-specific computational model of HGF/c-Met axis supports the findings of experimental studies reporting enhanced inhibition of phosphorylated AKT and ERK1/2 upon c-Met-α5β1 dissociation and highlights the importance of previously identified c-Met-β1 co-trafficking (Jafarnejad et al., 2019; Barrow-Mcgee et al., 2016).

## 5 Evidence of complex formation

Several of the studies reviewed here have reported the association of c-Met and integrin in a complex predominantly through co-IP and co-immunofluorescence experiments. Interestingly, complex formation is observed across the different signalling cooperation mechanisms.

### 5.1 c-Met-integrin association

c-Met-β1 (Hui et al., 2009; Bogorad et al., 2014; Singh et al., 2019), c-Met-α3 (Tesfay et al., 2016), and c-Met-α6β4 (Trusolino et al., 2001) association was shown to occur in a range of tumour cells in the absence of HGF. Other studies implicate the involvement of HGF in c-Met-integrin association, particularly with β1, α5β1, and αvβ3 integrins (Rahman et al., 2005; Huang et al., 2019b). Moreover, functional c-Met-integrin association has been described to occur at a basal level and to increase upon HGF stimulation (Mitra et al., 2011; Barrow-Mcgee et al., 2016; Jahangiri et al., 2017).

A detailed analysis using PyMOL modelling (Jahangiri et al., 2017) identified five amino acids of β1, at positions 246, 283, 284, 287, and 290, to be critical for c-Met binding. Two of these residues were further confirmed by site-directed mutagenesis (Lau et al., 2021). The binding sites on c-Met, however, remain unclear. It has been suggested that β4 integrin would interact at multiple different positions throughout c-Met as supported by β4 co-IP with a range of c-Met mutants (Trusolino et al., 2001). Moreover, a neutralising antibody of c-Met, which binds four amino-acid residues in its extracellular Sema domain, reduced the ability of several β1 integrin antibodies to detect their epitopes, suggesting a possible binding site on c-Met (Jahangiri et al., 2017). It is, however, unclear where exactly on c-Met integrin binding takes place.

The association of c-Met and β1 integrin detected by proximity ligation assay, which typically detects molecules at less than 40 nm distance, strongly suggests they interact directly (Barrow-Mcgee et al., 2016; Jahangiri et al., 2017). However, c-Met association with integrins may also occur in a complex with a range of other molecules. Most notably, tetraspanin transmembrane proteins, involved in cell-ECM interactions and integrin-GFR crosstalk, may play a role in facilitating integrin-c-Met cooperation by providing additional or alternative means of interaction. Thus, CD151 has been reported to occur as a part of c-Met and β4 or α3/α6 integrin complexes in gastric carcinoma (Franco et al., 2010) or salivary gland and breast cancer cells (Klosek et al., 2005; Klosek et al., 2009), respectively. Moreover, loss of CD151 upon siRNA-mediated KD disrupted c-Met-integrin complexes (Klosek et al., 2005; Klosek et al., 2009; Franco et al., 2010).

There are also reports of glycoprotein cluster of differentiation isoform six involvement in c-Met-α6β4 binding (Jung et al., 2011). c-Met-β1 complex with tensin-4 and c-Met-β4 complex with ErbB2 or sphingosine-1-phosphate receptor were also reported (Muharram et al., 2014; Yoshioka et al., 2013; Ephstein et al., 2013), whereas β5 has been shown to co-IP with c-Met and keratin 16 (Huang et al, 2019a). Upon β4 KD, β4-ErbB2-c-Met immunoprecipitate was shown to be lost (Yoshioka et al., 2013).

The induction or disruption of c-Met-integrin complex formation has functional consequences. The functional role of c-Met-β1 complex was further demonstrated with the engineering of an artificial heterodimerisation system where the rapamycin-derived drug AP21967 induces the heterodimerization of fused c-Met-FKBP (FK506-binding protein) and β1-FRB (FKBP-rapamycin-binding). The dimerization promoted wound healing and invasion of breast cancer cells (Jahangiri et al., 2017). A therapeutic humanised anti-β1 neutralising antibody strongly inhibited c-Met-β1 immunoprecipitation in breast cancer cells and reduced the mesenchymal phenotype of breast cancer cells (Jahangiri et al., 2017). Functionally, short-hairpin RNA KD of CD151 strongly reduced HGF-induced ERK1/2 phosphorylation and tumour growth (Franco et al., 2010). Wound healing of salivary gland cancer cells stimulated with HGF on Matrigel was also impaired upon CD151 or α3 or α6 siRNA (Klosek et al., 2005). Thus, collectively these findings indicate that the disruption of tetraspanin-c-Met-integrin complex has downregulatory effects on downstream signalling and tumorigenic cell behaviour.

### 5.2 Hepatocyte growth factor-integrin ligand association

Intriguingly, integrin-c-Met cooperation may also be mediated by the binding of HGF to an ECM integrin ligand, such as vitronectin or fibronectin, as seen in adhesive endothelial cells. HGF binding domains were identified on these two integrin ligands (Rahman et al., 2005). Thus, stimulation of endothelial cells with HGF and fibronectin or vitronectin, versus HGF alone, increased cell migration and proliferation. Downstream, c-Met, ERK1/2 and AKT phosphorylation and Ras-GTP activation were maintained longer and ERK1/2 or PI3K/AKT pharmacological inhibition impaired cell proliferation or migration. Cell migration was also inhibited by treatment with anti-α5β1 antibodies (Rahman et al., 2005). In contrast, HGF-fibronectin cooperativity is also exhibited by cells in anchorage-independent conditions, resulting in increased mammary carcinoma cell survival (Qiao et al., 2000). It could thus be proposed that HGF binding to integrin ligand could synergistically enable c-Met-integrin crosstalk.

## 6 Physiological and therapeutic relevance of c-Met-integrin cooperation

### 6.1 Evidence of cooperative signalling in tissues and *in vivo*

Although high levels of c-Met or integrin in patient tumour tissue have been shown to correlate with poor prognosis (Tjin et al., 2006; Sawada et al., 2007; Sawada et al., 2008), direct evidence of c-Met-integrin cooperation in human tissues and *in vivo* is scarce. Using proximity ligation assay one group has shown c-Met-integrin association in patient breast tumour tissue with elevated levels of c-Met-β1 complex in brain metastases compared to the primary tumours (Jahangiri et al., 2017). In addition, c-Met-β1 formation was reported at the invasive fronts of metastatic brain tumours of mouse xenografts compared to the primary breast tumour sites (Jahangiri et al., 2017).

The artificial heterodimerization of c-Met-FKBP and β1-FRB by the rapamycin-derived drug AP21967 promoted breast cancer cell extravasation out of circulation as shown through a tail vein metastasis assay (Jahangiri et al., 2017). c-Met-β1 complex induction was also associated with shorter mice survival, although there was no difference in gross metastases as detected by bioluminescence following intracardiac implantation of breast cancer cells (Lau et al., 2021).

One study reported a significant reduction of phosphorylated c-Met and total c-Met expression levels in HCC xenograft tumour tissue upon siRNA-mediated KD of β1, and a notable inhibition of HCC tumorigenic progression (Bogorad et al., 2014). Similarly, we have shown that HGF-driven *in vivo* tumorigenesis in mice and cell invasion in zebrafish embryos required the expression of β1 and the presence of its intact NXXY domain (Barrow-Mcgee et al., 2016). Although these experiments suggested the role of the signalling function of β1 in HGF-c-Met-driven *in vivo* invasion and tumorigenesis, *in vivo* evidence of an independence from β1 adhesive property was not provided. However, the requirement of the autophagy regulator autophagy-related gene five in the HGF-driven invasion of lung cancer cells in zebrafish was shown using siRNA, suggesting that the inside-in c-Met-β1 cooperation in ARE vesicles support the invasion (Barrow-Mcgee et al., 2016). Furthermore, there is *in vivo* evidence that c-Met-β4 integrin cooperation can occur independently from the adhesive property of integrin. Thus, cells expressing WT c-Met and extracellularly truncated β4 integrin resulted in the formation of tumours in mice, whereas cytoplasmic β4 mutants did not form tumour masses (Bertotti et al., 2005).

### 6.2 c-Met-integrin cooperation in drug resistance and the potential for combination therapy

As c-Met-integrin cooperation appears therapeutically important in diverse tumour contexts, it may be exploited in improving anti-tumour effects and modulating drug resistance. There has been a limited benefit of c-Met or integrin inhibition in the clinic (Mo and Liu, 2017; Huang et al., 2020; Li et al., 2021). Integrin inhibitors are antibodies (Alday-Parejo et al., 2019) which impair adhesive integrin function. They are therefore unlikely to block adhesion-independent role of integrins in regulation of c-Met signalling, which may contribute to the poor clinical benefit when used as a monotherapy. It would be interesting to investigate whether combining c-Met and integrin inhibitors would lead to a better response than using each drug as a monotherapy. An alternative strategy could be to target c-Met and molecules downstream of the integrin. Indeed, FAK activation associated with α5 upregulation in response to c-Met inhibitor cabozantinib appears to reduce its anti-tumour effects, whereas combination of cabozantinib with FAK inhibitor CT-707 improves therapeutic activity as shown through increased apoptosis *in vitro* and reduced tumour growth in HCC xenografts (Wang et al., 2016).

Further, integrins and c-Met cooperation may be a potent mediator of resistance to therapeutics modulating the behaviour of other molecules (Cruz Da Silva et al., 2019). Thus c-Met-β1 complex was reported to promote glioblastoma cell migration and local invasion as part of a resistance mechanism to bevacizumab, an anti-angiogenic VEGF monoclonal antibody. As VEGF bound to VEGF receptor two appeared to sequester c-Met and β1, bevacizumab therapy, which inhibits VEGF, may result in c-Met-β1 complex formation and its driven invasion (Jahangiri et al., 2017).

Histone deacetylase inhibitor suberanilohydroxamic acid reportedly leads to an upregulation of α5β1 which mediates c-Met phosphorylation associated with poor sensitivity in prostate and lung cancer cells *in vivo* (Ding et al., 2015). Although the means of α5β1-c-Met cooperation were not elucidated, α5β1 KD led to a significant reduction in c-Met phosphorylation. Thus, c-Met inhibition or lack of α5β1 improves the sensitivity to suberanilohydroxamic acid, which indicates that the manipulation of c-Met or an integrin may enhance effectiveness of other targeted therapeutics.

## 7 Discussion

**FIGURE 5:**
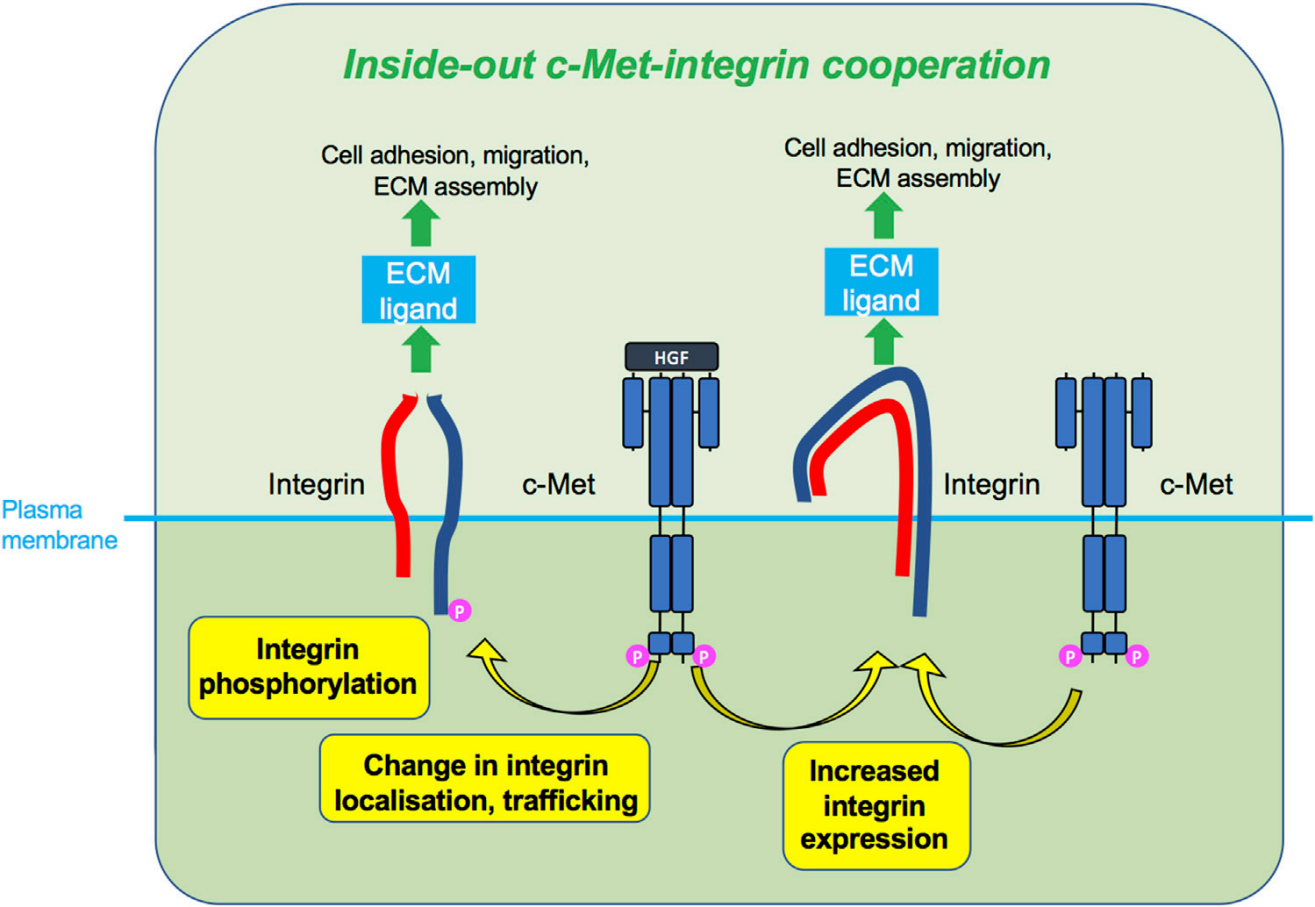
Inside-out c-Met-integrin cooperation. c-Met, upon HGF stimulation or sometimes without HGF, promotes integrin activity, such as phosphorylation, increased expression or change in localisation. Activated integrins can in turn bind ECM ligand and induce cellular responses such as adhesion and migration.

**FIGURE 6:**
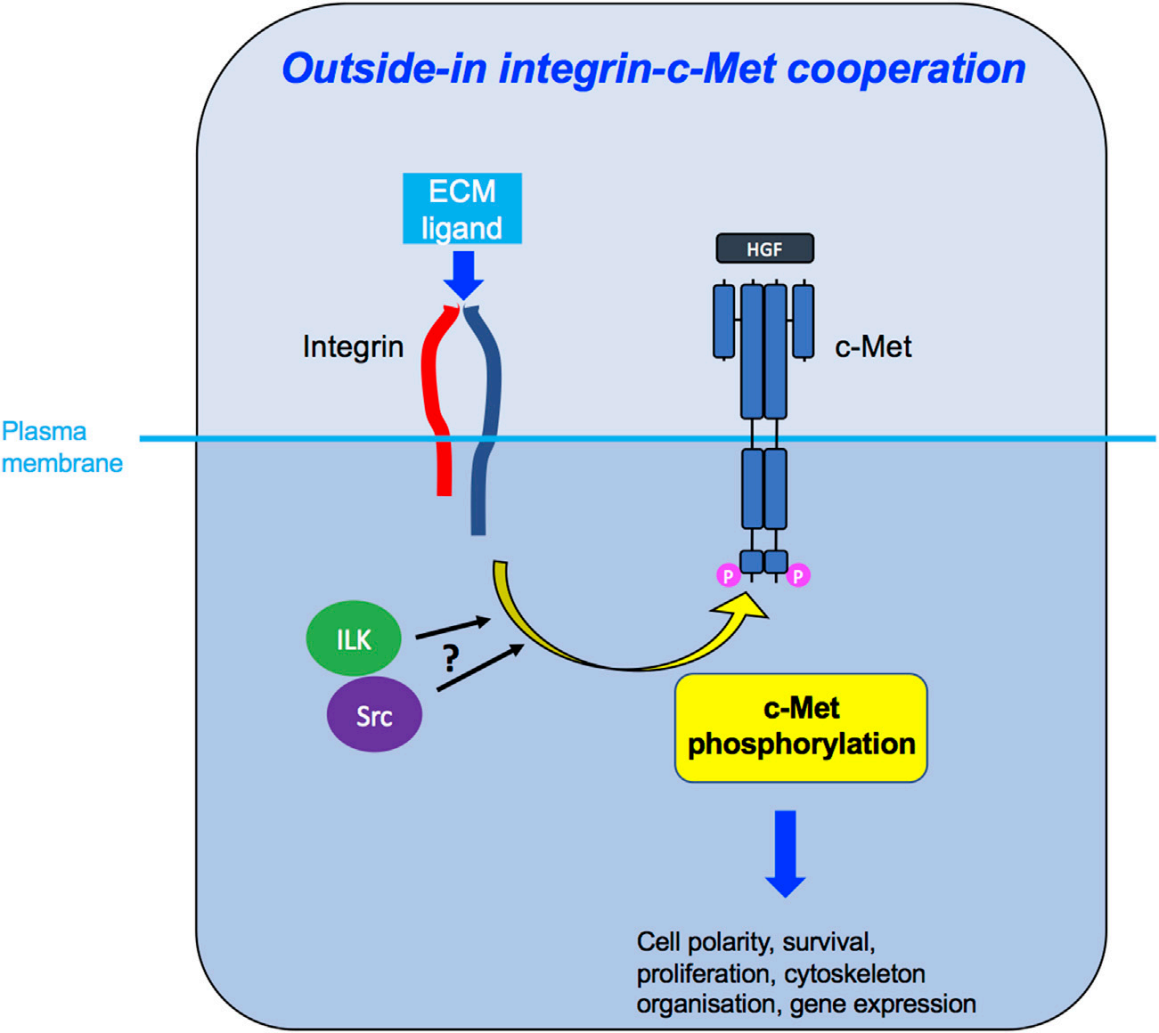
Outside-in integrin-c-Met cooperation. Integrin activation, through binding to its ligand in the ECM, leads to downstream c-Met phosphorylation, potentially *via* ILK and/or Src kinases, enabling c-Met-induced intracellular signalling and diverse cellular functions.

**FIGURE 7:**
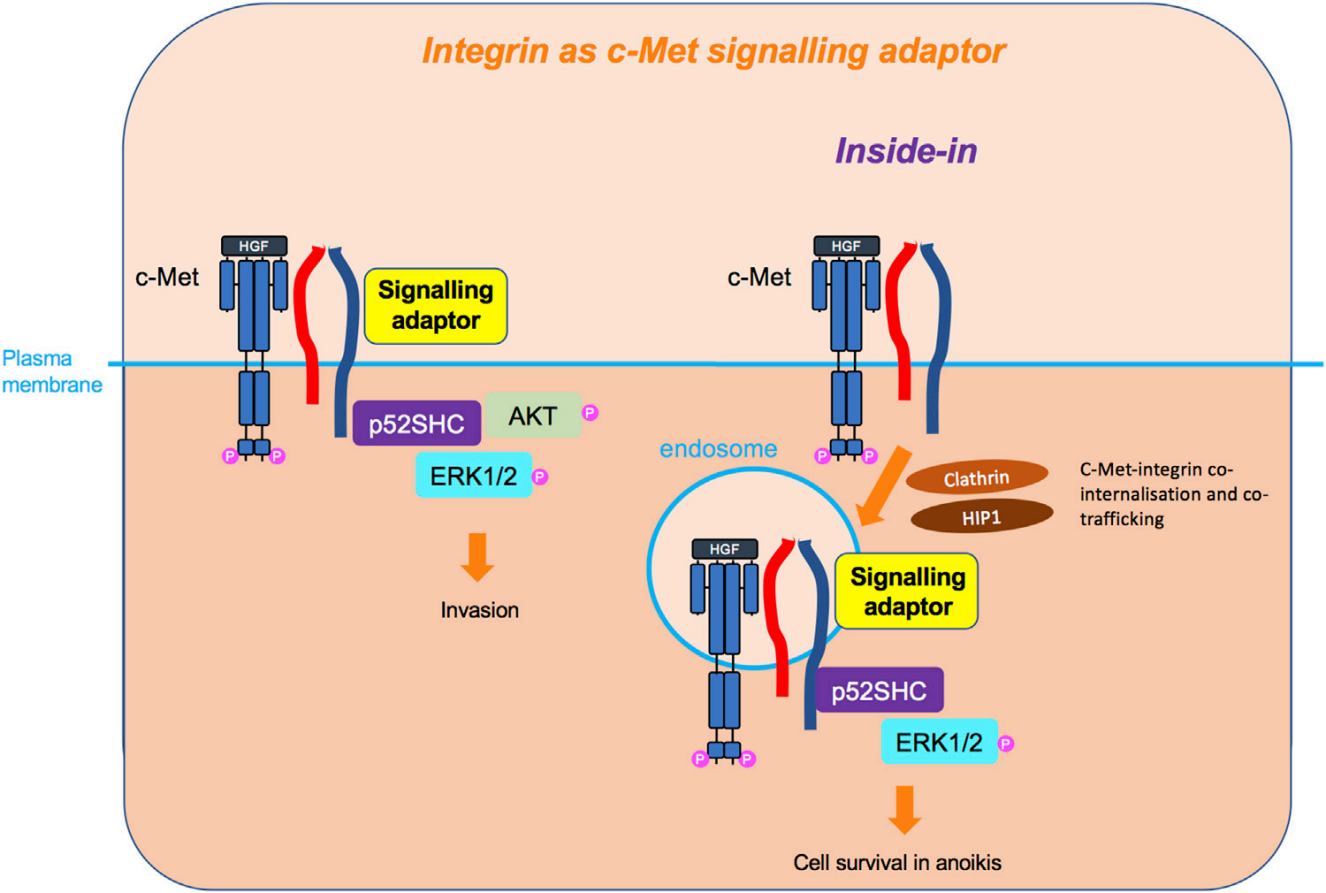
Integrin as c-Met signalling adaptor and the inside-in signalling. Integrins, so far β4 and β1, can also act as adaptors independently of their extracellular domain and adhesive property, linking c-Met to downstream signalling molecules, such as p52SHC adaptor, leading to ERK1/2 and AKT sustained signalling. For the β1 integrin, this cooperation has been shown to occur on an endomembrane, following the co-internalisation of c-Met and β1. This inside-in signalling triggers cell survival independently of anchorage.

Having reviewed in detail the literature on c-Met and integrin cooperation, we propose that the two molecules utilise three main mechanisms of signal integration which include inside-out (Figure 5) and outside-in (Figure 6) signalling, in an HGF-dependent or -independent manner. Both mechanisms engage integrin adhesion to its extracellular ligand. The third mode of cooperation (Figure 7), less conventional, is the role of an integrin as a signalling adaptor to amplify c-Met signalling, which appears to be independent from the adhesion function of integrins. Moreover, there is evidence that such cooperation can occur on endosomes, enabling an inside-in signalling, instead of the classical view of membrane receptors signalling at the plasma membrane. Some of the more recent studies revealing spatially mediated c-Met-integrin interactions indicate greater complexity than the regulation of expression levels or activity.

Although our review highlights these three main mechanisms of cooperation, they are not necessarily mutually exclusive and a few studies have shown elements of inside-out and outside-in c-Met-integrin cooperation in the same cells (Jahangiri et al., 2017; Sridhar and Miranti, 2006; Ephstein et al., 2013; Li et al., 2013).

Although the studies reviewed here show some elements of evidence for mainly c-Met-β1 cooperation, many did not dissect the mechanisms of cooperation including whether the two molecules form heterodimers. Therefore, the level of specificity and mechanistic diversity of c-Met-integrin cooperation remains poorly defined. In light of the studies reporting spatial facilitation and regulation of cooperation predominantly between c-Met and β1, we believe that the true scope and importance of spatially controlled c-Met interaction with a range of integrins is yet to be revealed.

**FIGURE 8:**
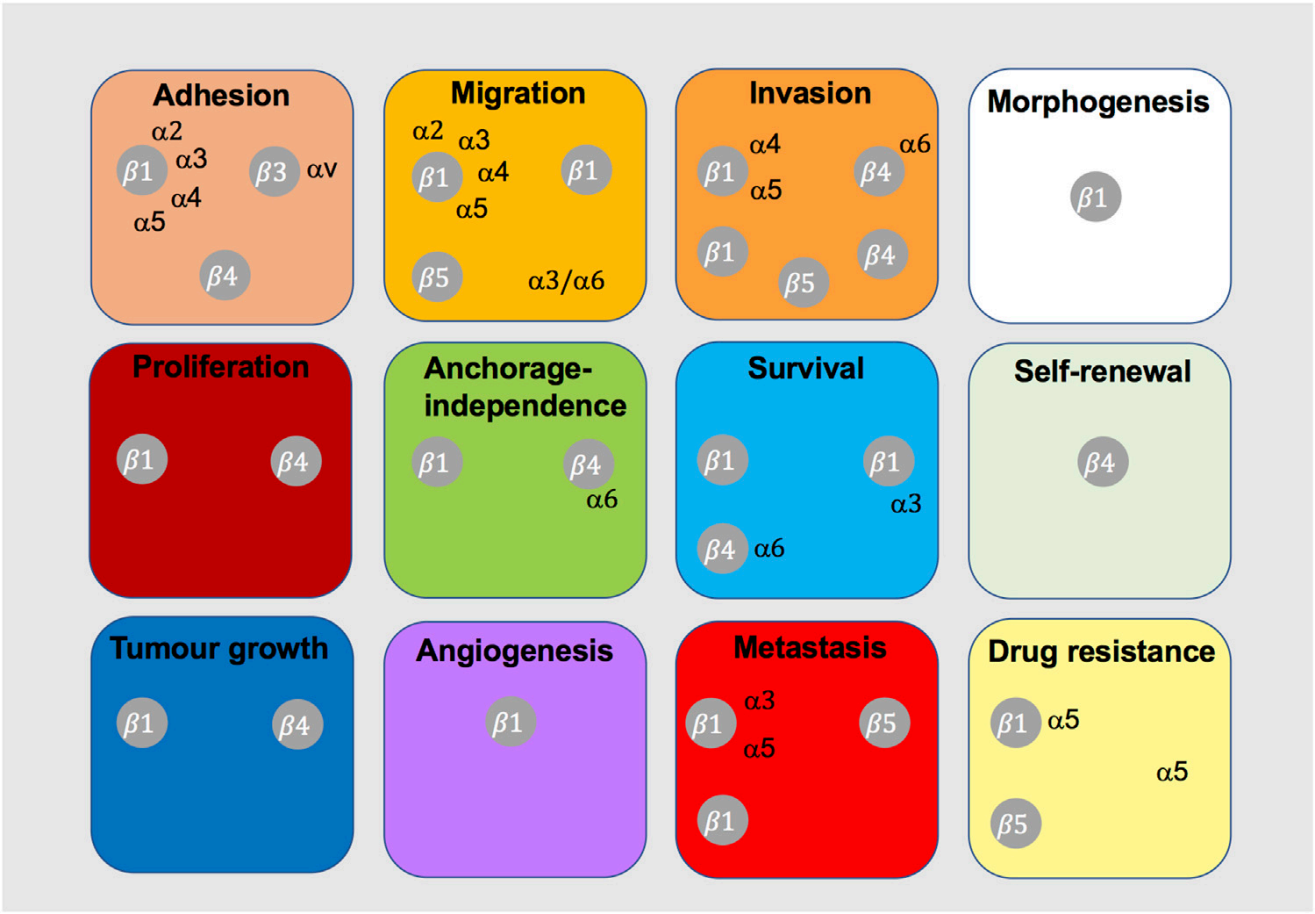
Integrins shown to cooperate with c-Met in tumorigenic cell behaviour. This diagram indicates the integrins that have been shown to cooperate with c-Met for the indicated cancer-related cell function or process.

Signalling events induced by c-Met-integrin cooperation are poorly defined, with limited evidence on the exact signalling mechanisms that induce tumorigenic functions. Interestingly, c-Met-integrin cooperation appears to contribute to more global changes in transcriptional cell profiles (Lau et al., 2021), potentially suggesting yet unexplored signalling networks and downstream effects. Phenotypically, it is evident that c-Met-integrin cooperation contributes to diverse cancer cell functions (Figure 8), particularly migration, invasion and anchorage-independent growth or survival. These phenotypes are believed to promote metastasis, as well as drug-resistance, across different tumour types.

Mechanical ECM properties may also regulate c-Met-integrin crosstalk. ECM stiffness may modulate c-Met-β1 interaction, as increased matrix rigidity results in upregulated c-Met, β1 and FAK, and associated cell proliferation, epithelial-mesenchymal transition and resistance to tyrosine kinase inhibitors (Chang et al., 2015). Differential and dynamic regulation of integrins with oncogenic c-Met may facilitate both early neoplastic growth and later invasion and metastasis, key in disease progression.

Other oncogenic partners may enhance c-Met-integrin cooperation as exhibited by mutant NRP1 co-receptors (Huang et al., 2019b). Moreover, HGF/c-Met axis induced integrin-mediated collective mesenchymal migration while constitutively active c-Met led to an integrin-independent cell rounding, suggestive of amoeboid migration (Mai et al., 2014). It thus could be postulated that the presence or absence of c-Met-integrin association in some contexts may mediate different types of cell migration. Importantly, however, *in vitro* cell migration and invasion models do not fully recapitulate the physiological conditions, posing a challenge to understand the specific functional role integrin-c-Met interaction may play in the tumour microenvironment. Encouragingly, evidence of viable therapeutic manipulation of c-Met-integrin signalling systems *in vivo* suggests potential exploitation of their cooperation which could enable more effective treatment strategies than c-Met or integrin inhibitors used as a monotherapy.

Therefore, we must strive for a greater understanding of c-Met-integrin cooperation, aiming to thoroughly elucidate their signalling networks and the associated tumorigenic effects. Understanding the mechanisms of cooperation, signalling cascades and their functional effects on tumour cell behaviour in a tumour microenvironment context would potentially provide novel therapeutic avenues direly needed to improve clinical outcomes.
